# Editorial: The IL-17 Cytokine Family in Tissue Homeostasis and Disease

**DOI:** 10.3389/fimmu.2021.641986

**Published:** 2021-02-24

**Authors:** Nicola I. Lorè, Kong Chen, Katarzyna Bulek

**Affiliations:** ^1^Division of Immunology, Transplantation, and Infectious Diseases, Emerging Bacterial Pathogens Unit, IRCCS San Raffaele Scientific Institute, Milan, Italy; ^2^Università Vita-Salute San Raffaele, Milan, Italy; ^3^Division of Pulmonary, Allergy, and Critical Care Medicine, Department of Medicine, University of Pittsburgh, Pittsburgh, PA, United States; ^4^Department of Immunology, Jagiellonian University, Kraków, Poland; ^5^Department of Inflammation and Immunity, Cleveland Clinic, Cleveland, OH, United States

**Keywords:** IL-17 cytokine family, host-pathogen, cancer, autoimmunity, inflammatory disease

The IL-17 cytokine family represents a wide class of pleiotropic inflammatory molecules that are structurally related. The IL-17 cytokines can modulate complex dynamic interactions between stromal and immune cells and determine the outcome of pathophysiological processes. Historically the most well-known cytokines across the IL-17 family are the IL-17A, IL-17F and IL-17E (also known as IL-25) while others such as IL-17B, IL-17C or IL-17D are emerging in modulating tissue homeostasis and disease (1). This cytokine family activates downstream signaling through the IL-17 receptor (IL-17R) family, which includes five members named IL-17receptor(R)A, IL-17RB, IL-17RC, IL-17RE, and IL-17RD (1, 2). In this context, IL-17RA can play a pleiotropic role by interacting with other IL17 receptors, including IL-17RC, IL-17RB, IL-17RE, and IL-17RD.

In this Research Topic you will find a number of original articles and reviews aiming at shedding light on the multifaceted role of IL-17 cytokines and their receptors in the field of immunity, host-pathogen interactions, autoimmunity and tumor immunology.

The mini review by Brevi et al. describes the role of the IL-17 family cytokines in the interplay between microbiota and epithelial cells that may contribute to the cancer pathogenesis. The authors purposely focused on IL-17B-to-F, which role is less understood, and discussed differences and similarities between these cytokines in the microbiota-immunity-cancer axis. Better understanding of these relationships may provide therapeutic strategies targeting IL-17-related diseases.

The emerging role of the IL-17B/IL-17RB axis in cancer has been discussed in review article of Bastid et al. with a particular attention on tumorigenesis and resistance to anticancer therapies. They described the expression and signaling pathways of the IL-17B/IL-17RB axis such as cellular sources and its role in inflammatory disease. They clearly highlighted how several reports proposed the potential role of IL-17B or its receptor in the outcome of different cancer types, such as breast carcinoma, gastric cancer, lung cancer, primary glioblastoma, lymphomas or acute myeloid leukemia. Moreover, they deeply described potential mechanisms of action by the IL-17B/IL-17RB axis not only in enhancing the proliferative, migratory and invasive properties of tumor cells, but also in impairing the anti-tumor immune response and favoring resistance to cancer treatments.

In the review article, Nies and Panzer discussed the latest discoveries about the identification, regulation, and function of the IL-17C/IL-17RE pathway. The authors described the mechanisms of IL-17C/RE driven inflammation in epithelial and Th17 cells and discussed its role in the context of infectious and autoimmune diseases. They summarized the role of the axis in bacterial, fungal, and viral infections. Moreover, they reviewed the first approaches to target IL-17C/IL-17RE axis, which they believed would be especially important for the treatment of autoimmune disorders.

The study of Adams et al. shows the generation and the characterization of a novel humanized IgG1 antibody, named Bimekizumab, able to neutralize both IL-17A and IL-17F cytokines. In this context we can speculate that strategy to block both IL-17A and IL-17F cytokines may be useful as therapeutic option in diseases where the treatment anti IL-17RA failed. It's worth noting that the block of IL-17RA signaling may alter other inflammatory pathways modulated by other IL-17 receptors (e.g., IL-17RB or IL-17RC) and independent by IL-17A or IL-17F cytokines. This new monoclonal neutralizing antibody represents a new potential therapeutic strategy when both IL-17A and IL-17F contributes to disease progression.

*Porphyromonas gingivalis* can cause oral microbiome dysbiosis and contributes to the development of periodontitis. Using a mouse model, Bittner-Eddy et al. show that persistent oral *P. gingivalis* infection initiated an IL-17A-biased response dominated by Th17 cells and a distinct population of IL-17A-expressing Treg cells that changes into a late Th1 response with only sporadic phenotypic conversion from Th17 cells. Understanding the mechanism of Treg-Th17 transdifferentiation may provide novel targets to control the inflammatory disease processes.

Among the different potential biological function of IL-17A, Ramakrishnan et al. shows that IL-17A cytokine increases mitochondrial dysfunction in primary asthmatic bronchial fibroblasts. Moreover, IL-17 increased the expression of autophagy-related genes to a statistically significant extent in severe asthmatic fibroblasts than in healthy, suggesting a unique response to IL-17 stimulation in fibroblasts from patients with severe asthma. Overall, the data presented in this work suggest that IL-17 may be considered a potent inducer of pro-fibrotic phenotype through induction of autophagy in bronchial fibroblasts.

Chronic inflammation in obesity is believed to be associated with severity of asthma and Th17 cells have been shown to be associated with severe asthma due to their resistance to steroid treatment. Heialy et al. examined adipocytes responses to Th17 cytokines from lean and obese subjects and found out not only that stimulation leads to further inflammation in adipocytes obtained from obese subjects, but also that IL-17 may modulate adipocyte responses to steroids and obese adipocytes are not responsive to steroid treatment. Indeed, serum obtained from obese and morbidly obese asthmatic patients showed a significant decrease in GR-α/GR-β ratio, a marker for steroid resistance in adipocytes, and an increase in IL-17F and IL-13 compared to lean and overweight patients. To a certain extent, this study explains why steroid hyporesponsiveness is commonly described in obese asthmatics.

The mini review article of Capone and Volpe shed the light on the transcriptional regulators of T-helper 17 Cell differentiation in health and autoimmune diseases. The authors focused their attention on transcription factors modulating the levels of retinoic acid-related orphan nuclear receptors and IL-17A, with a particular attention on Th-17 population. The potential involvement of Th17-related transcriptional regulators has been deeply described in the context of autoimmune diseases, such as crohn's disease and multiple sclerosis. They concluded with an interesting overview on the potential therapeutic approaches of targeting transcriptional regulators of Th17 cells in experimental model of “autoimmune diseases” and clinical trials.

In the review article of Milovanovic et al., the critical role for IL-17 and T helper 17 cells have been discussed in the pathogenesis of chronic inflammatory and autoimmune diseases. In this context, the authors review and discussed the biological processes related to IL-17A signals and Th17 cells with a particular attention on environmental factors influencing the pathogenic potential of Th17 cells. Of interest they reviewed and discussed literature related to IL-17 cytokine and the cellular target as therapeutic approach in multiple sclerosis, Alzheimer's disease and ischemic brain injury. Overall this review article shows an interesting overview related to the role of IL-17 and its therapeutic potential targeting in the pathogenesis of neuroinflammatory and neurodegenerative diseases.

The research article of Chen et al. demonstrates that mTOR blockade can inhibit IL-17A and TNFα production and suggest that mTOR targeting may support an alternative therapeutic option in fighting of progression of spondyloarthritis disease. In particular, the targeting of mTOR is beneficial to inhibit IL-17A and TNFα protein production by human peripheral blood mononuclear cells from spondyloarthritis patients and to reduce IL-17A expression in inflamed joints using HLA-B27 tg rat model.

Cutaneous squamous cell carcinoma (cSCC) is the second most common type of non-melanoma skin cancer and genome-wide association study for cSCC suggests a role for aryl hydrocarbon receptor (AhR) and IRF4. Sato et al. investigated the role of AhR signal in keratinocytes for the development of cSCC using a two-stage chemically induced skin carcinogenesis mouse model and human cSCC samples. The authors showed that AhR ligands increase the expression of IL-17 downstream genes in normal human epidermal keratinocytes; the number of cutaneous SCC lesions is decreased in AhR deficient mice; and in patients' samples, atypical keratinocytes overexpress Th17 downstream genes in tumor lesions of cSCC as compared to normal keratinocytes at the marginal zone of the tumor. These data support the hypothesis that AhR ligands promote the development of cSCC through induction of Th17 cells.

Overall, this special topic highlights recent advances for a better understanding of IL-17 cytokine family in tissue homeostasis and disease. Here, we wanted to show the broad biological function mediated by IL-17 cytokine family ranging from immune defense against pathogens to the modulation of inflammation and tumor immunology, as observed in infection, inflammatory diseases or cancer. Moreover, several therapeutic approaches targeting the IL-17 cytokine family are available and they are paving the way for the development of specific strategies limiting the progression of different diseases including autoimmunity, chronic immune-mediated diseases, lung illnesses, and tumor immunology.

## Author Contributions

NL, KC, and KB edited the topic and wrote the manuscript. All authors contributed to the article and approved the submitted version.

## Conflict of Interest

The authors declare that the research was conducted in the absence of any commercial or financial relationships that could be construed as a potential conflict of interest.
